# Insight into the Interactions between Novel Isoquinolin-1,3-Dione Derivatives and Cyclin-Dependent Kinase 4 Combining QSAR and Molecular Docking

**DOI:** 10.1371/journal.pone.0093704

**Published:** 2014-04-10

**Authors:** Junxia Zheng, Hao Kong, James M. Wilson, Jialiang Guo, Yiqun Chang, Mengjia Yang, Gaokeng Xiao, Pinghua Sun

**Affiliations:** 1 Faculty of Chemical Engineering and Light Industry, Guangdong University of Technology, Guangzhou, P. R. China; 2 Department of Medicinal Chemistry, College of Pharmacy, Jinan University, Guangzhou, P. R. China; 3 Department of Drug Discovery, H. Lee Moffitt Cancer Center and Research Institute, Tampa, Florida, United States of America; 4 College of Pharmacy, University of South Florida, Tampa, Florida, United States of America; Russian Academy of Sciences, Institute for Biological Instrumentation, Russian Federation

## Abstract

Several small-molecule CDK inhibitors have been identified, but none have been approved for clinical use in the past few years. A new series of 4-[(3-hydroxybenzylamino)-methylene]-4H-isoquinoline-1,3-diones were reported as highly potent and selective CDK4 inhibitors. In order to find more potent CDK4 inhibitors, the interactions between these novel isoquinoline-1,3-diones and cyclin-dependent kinase 4 was explored via in silico methodologies such as 3D-QSAR and docking on eighty-one compounds displaying potent selective activities against cyclin-dependent kinase 4. Internal and external cross-validation techniques were investigated as well as region focusing, bootstraping and leave-group-out. A training set of 66 compounds gave the satisfactory CoMFA model (*q* ^2^ = 0.695, *r* ^2^ = 0.947) and CoMSIA model (*q* ^2^ = 0.641, *r* ^2^ = 0.933). The remaining 15 compounds as a test set also gave good external predictive abilities with *r* ^2^_pred_ values of 0.875 and 0.769 for CoMFA and CoMSIA, respectively. The 3D-QSAR models generated here predicted that all five parameters are important for activity toward CDK4. Surflex-dock results, coincident with CoMFA/CoMSIA contour maps, gave the path for binding mode exploration between the inhibitors and CDK4 protein. Based on the QSAR and docking models, twenty new potent molecules have been designed and predicted better than the most active compound **12** in the literatures. The QSAR, docking and interactions analysis expand the structure-activity relationships of constrained isoquinoline-1,3-diones and contribute towards the development of more active CDK4 subtype-selective inhibitors.

## Introduction

Cyclin-dependent kinases (CDKs), a family of serine/threonine protein kinases, play a central role in the growth, development, proliferation and death of eukaryotic cells [1], [2]. There are more than 13 CDKs of which 12 different cyclin families have been identified up to now, and different CDK/cyclin combinations are active during each phase of the cell cycle [3]–[5]. Among these CDK/cyclin complexes, the D/CDK4 and E/CDK2 complexes have been greatly concerned [6]. In the G1-S phase transition, the retinoblastoma susceptibility gene family of proteins (Rb) was phosphorylated by the D-type cyclins (D1, D2 or D3) in combination with CDK4 and cyclin E/CDK2 complexes. Phosphorylation of the Rb activated the E2F transcription factors and resulted in the transcription of genes required for DNA synthesis. This kind of function exerted by D/CDK4 and E/CDK2 complexes is positively regulated by the mitogenic signaling pathways and negatively regulated by the cyclin-dependent kinase inhibitors (CKIs) [7]–[13]. Inhibition of cyclin-dependent kinases (CDKs) with small molecules has been suggested as a strategy for treatment of cancer, based on deregulation of CDKs commonly found in many types of human tumors. Selective CDK inhibitors such as CYC-202 [14] and BMS-387032 [15] targeting CDK2, and PD0332991 [16] targeting CDK4 have been under clinical evaluations. Recently, a series of novel isoquinoline-1, 3-(2*H*, 4*H*)-diones have been found to possess excellent selective inhibitory activity against the CDK4 [7], [8].

The three-dimensional quantitative structure-activity relationship (3D-QSAR) models derived from the most widely used computational methods, CoMFA (comparative molecular field analysis) and CoMSIA (comparative molecular similarity indices analysis), could be used to guide rational synthesis of potent novel inhibitors and now aimed to elucidate the structural features required for CDK4 inhibitors. The best developed models have been duly validated by a systemic external validation, on the basis of which a set of twenty new potent molecules have been designed and predicted stronger activity than **12,** the most active compound reported in the literatures[7], [8].

**Figure 1:**
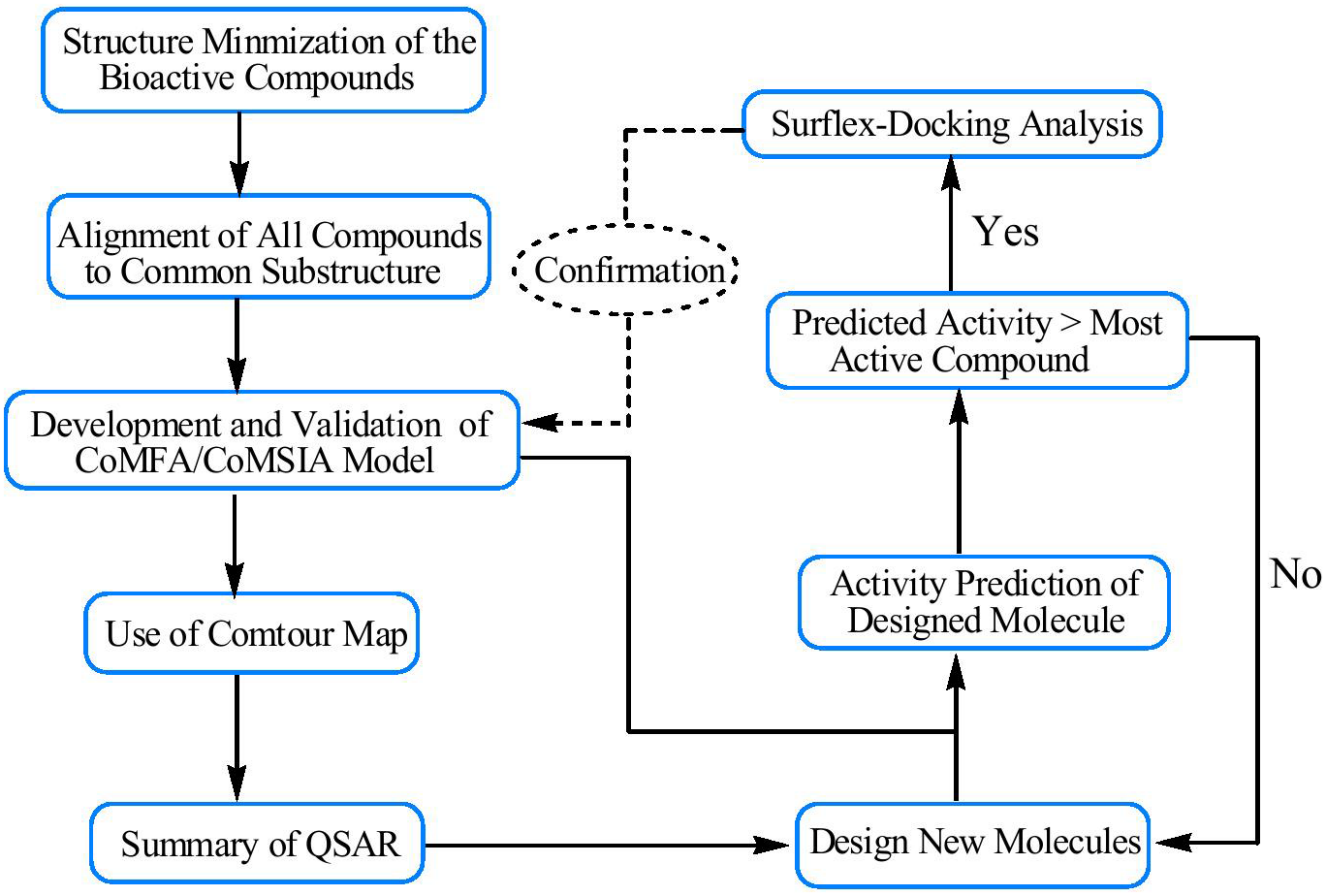
Step-wise description ofmethodology used for 3D-QSAR analysis, molecular docking and designing of new inhibitors for CDK4.

Molecular docking techniques have been extensively used as an important tool in the discovery of new small-molecule drugs for targeting proteins.[17]–[21] Based on an idealized representation that a ligand makes every potential interaction with the binding sites, docking uses an incremental construction algorithm to place flexible ligands into a fully specified active site. Surflex-Dock is particularly successful at eliminating false positive results and therefore used to narrow down the screening pool significantly, while retaining a large number of active compounds. The binding interactions of the isoquinolinedione derivatives within the CDK4 active sites were discussed. The step-wise description of methodology used for 3D-QSAR analysis, molecular docking and designing of new CDK4 inhibitors is as shown in Figure 1.

## Materials and Methods

### Data Set

**Figure 2:**
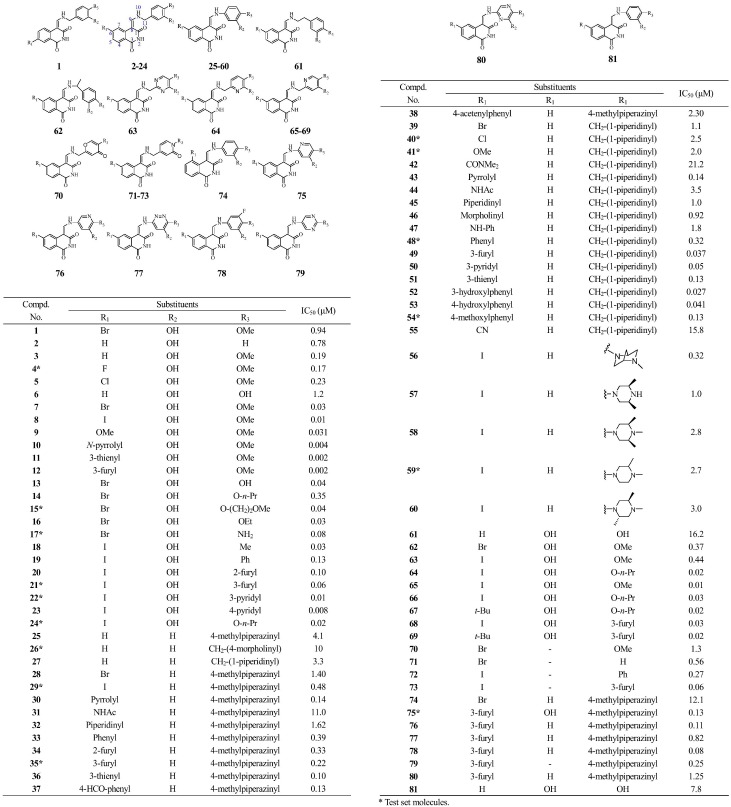
Chemical structures and IC50 values of the training and test set molecules.

All isoquinoline-1,3-(2*H*,4*H*)-dione derivatives and their biological activities were collected from literatures [7], [8] (Figure 2). A total set of 81 molecules were randomly segregated into training and test sets comprising 66 and 15 molecules, respectively, based on the following rules: (i) Diversity of the molecules was very necessary to assess the statistical significance. (ii) To avoid any redundancy or bias in terms of structure features and activity range, the information of the selected compounds must be clear and concise. (iii) The most active and least active compounds should be included in the training set. The activities of the CDK4 inhibitors were reported in IC50 and converted to pIC50 by taking Log (1/IC50) for the convenience. The activity range from 4.6 to 8.6 log units of these compounds provided a broad and homogenous data set for 3D-QSAR study. In general, the spread of activity should cover at least 3 log units for a reliable 3D-QSAR model [22].

### Molecular Modeling and Alignment

**Figure 3:**
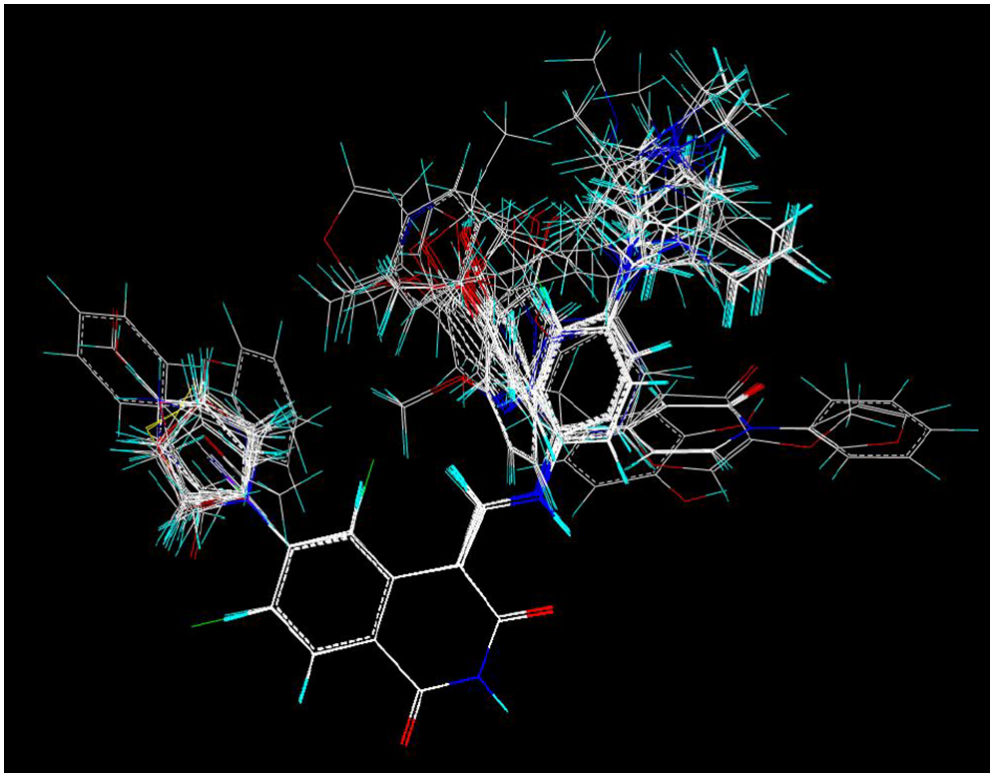
Alignment of the compounds used in the training set.

The structures of the derivatives were sketched in SYBYL 8.1 (Tripos, Inc., St. Louis, MO, USA) molecular modeling package and Gasteiger-Hückel charges were assigned to the atoms of all the compounds. A good alignment is the most essential for the quality and the predictive ability of CoMFA and CoMSIA models [23], and common substructure, pharmacophore or docking overlaps can be available to align molecules [24], [25]. The isoquinoline-1, 3-(2H, 4H)-dione ring with structural rigidity was selected as the common substructure and the compound **12** with the strongest inhibitory activity as the template molecule (Figure **3**). It can be seen that all the compounds studied have similar active conformations.

### CoMFA and CoMSIA Setup

Three-dimensional grid spacing was set at 2 Å in the x, y, and z directions. The steric and electrostatic field energies were calculated using the Lennard-Jones and the Coulomb potentials [26]. For CoMFA method, a *sp* ^3^ hybridized carbon atom with a+1 charge was identified as the probe atom to determine the magnitude of the steric and electrostatic field values, whose truncation was set at 30 kcal/mol [27]–[29].

The CoMSIA method, similar to CoMFA in terms of fields around the molecule, was based on the assumption that changes of ligands in binding affinities are associated with changes of molecular properties. Besides steric and electrostatic fields, three other different fields of hydrophobic, hydrogen bond donor and hydrogen bond acceptor are also calculated [30]. Moreover, a Gaussian function introduced in similarity indices makes it be calculated at all grid points, inside and outside different molecular surfaces. Equation used to calculate the similarity indices is as follows:
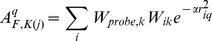
Where, *A* is the similarity index at grid point *q*, summed over all atoms *i* of the molecule *j* under investigation. *W*_probe, k_ is the probe atom with radius 1 Å, charge +1, hydrophobicity +1, hydrogen bond donating +1 and hydrogen bond accepting +1. *W*_ik_ is the actual value of the physicochemical property *k* of atom *i*. *r*_iq_ is the mutual distance between the probe atom at grid point *q* and atom *i* of the test molecule. α is the attenuation factor whose optimal value is normally between 0.2 and 0.4, with a default value of 0.3 [31].

### Partial Least Squares (PLS) Analysis

For Partial Least Squares (PLS) analysis [32], the “leave-one-out” cross-validation method was first carried out to generate a cross-validated *r* ^2^ (*q* ^2^) value and the optimal number of components (ONC), based on the lowest standard error of prediction (SEP) which usually corresponds to the highest cross-validated squared coefficient (*q* ^2^). To avoid over-fitting the models, a higher component was accepted only when the *q* ^2^ differences between two components was larger than 10% [24]. Region focusing was performed to maximize *q* ^2^ value by rotating the extracted principal components [33]. The *q* ^2^ is a good indicator of the accuracy of actual predictions and a *q* ^2^ value of 0.5 means halfway between no model and a perfect model [34]. Non-cross-validation was then executed to establish the final 3D-QSAR model after the optimal number of components was determined. The consequential final PLS models gave the conventional correlation coefficient (*r* ^2^), standard errors of estimate (SEE) and *F* ratio between the variances of calculated and observed activities. The equation for SEE is given below
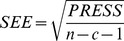
Where, n and c is the number of compounds and components, respectively, and PRESS is the sum of squared deviations between predicted and actual activity values for each molecule in the test set.

### External Validation

*q* ^2^ is often a useful but not sufficient criterion for model validation. In many cases, a model with high *r^2^_cv_* and *r* ^2^ values were proved to be inaccurate. Even though a model may exhibit a good predictive ability based on the statistics for the test set, it is not always sure that the model will perform well on a new set of data [35]. Therefore, an external test sets (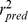) [36] was recommended for the estimation of predictive ability. Predictive values 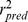 were calculated as follows:
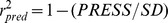
Therein, SD is the sum of squared differences between the measured activities of the test set and the average measured activity of the training set.

Several other statistics such as *r* ^2^_m_, *r* ^2^_0_, R and k were calculated using the following equations, and 3D-QSAR models were considered acceptable only if they satisfy the following conditions: *r^2^_cv_*>0.5, *r^2^*>0.6, [(*r^2^−r_0_^2^*)/*r^2^*] <0.1, 0.85≤k≤1.15 and *r* ^2^_m_>0.5 [36], [37].
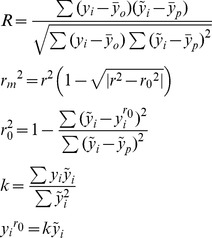
Where, *y_i_* and 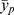 are the actual and predicted activities, respectively. 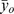 and 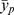 are the average values of the observed and predicted pIC_50_ values of the test set molecules, respectively. *r^2^* is the non-cross-validated correlation coefficient from PLS process.

### Molecular Docking

Surflex-Dock in SYBYL 8.1, using a patented search engine and an empirical scoring function to dock ligands into a protein’s binding site [19], was applied to study molecular docking in the present paper. The crystal structure of CDK4 with ligand 1GIH was retrieved from the RCSB Protein Data Bank [38]. A protomol, a computational representation of the receptor’s binding cavity to which putative ligands are aligned, was generated automatically with a threshold parameter of 0.31 and a bloat parameter of 1 Å, and composed of a collection of fragments or probe molecules such as CH4, N-H, and C = O that characterize steric effects in the binding pocket, hydrogen bond donor and acceptor groups, respectively.[39], [40] All the water molecules and sulfate salt in CDK4 1GIH (receptor) were deleted, and hydrogen atoms and Gasteiger charges were added [41], [42]. All of the eighty-one ligands were docked sequentially into the binding pocket of CDK4 using the parameters previously optimized. Surflex-Dock total scores are expressed in log_10_(*K_d_*) to represent binding affinities. The scores of 10 docked conformers of each isoquinolinedione derivatives were ranked in a molecular spreadsheet, and the highest total score was taken into consideration for ligand-receptor interactions. To visualize the binding mode between the protein and ligands, the MOLCAD (Molecular Computer Aided Design) program was applied to calculate and display the surfaces of channels and cavities, as well as the separating surface between protein subunits. MOLCAD program provides several types to create a molecular surface, in which the Robbin surfaces illustrating the secondary structure elements of the binding structure was applied to build the MOLCAD Robbin and Multi-Channel surfaces displayed with several potentials. Other parameters were established in default.

## Results and Discussion

### CoMFA and CoMSIA Analysis

**Figure 4:**
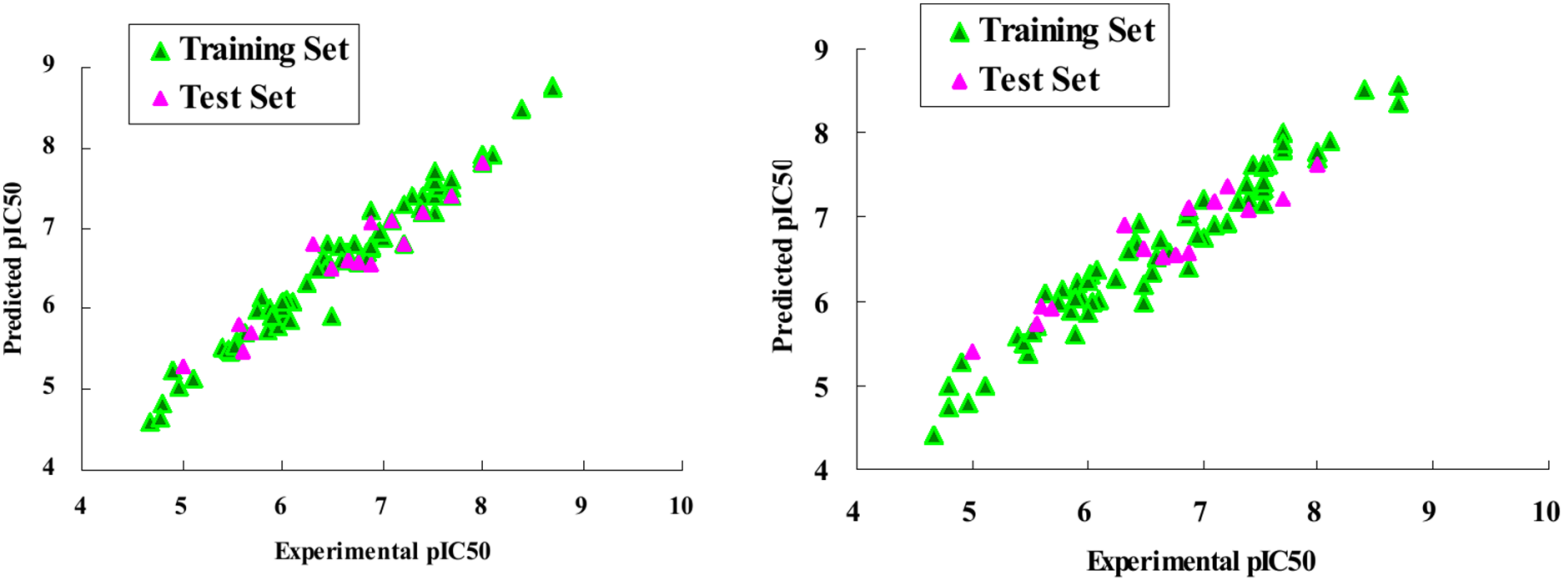
Graph of actual versus predicted pIC_50_ of the training set and the test set using CoMFA (Left) and CoMSIA (Right).

The results taken from the PLS analysis were summarized in Table **1**. The actual versus the predicted pIC_50_ values for the training and the test set molecules were listed in Table **2** and depicted graphically in Figure 4. For the CoMFA model after region focusing, the leave-one-out cross-validated *q* ^2^ value was 0.695 (>0.5) and non-cross-validated *r* ^2^ value was 0.947 with an optimized component of 6, standard error estimate (SEE) of 0.185 and *F* value of 139.423. Contributions of steric and electrostatic fields were 0.479 and 0.521, respectively.

**Table 1:** PLS results of CoMFA and CoMSIA models.

	CoMFA(before region focusing)	CoMFA (after region focusing)	CoMSIA
*PLS* statistics			
LOO cross *q* ^2^/SEP	0.544/0.727	0.695/0.348	0.641/0.496
Group cross *q* ^2^/SEP	0.578/0.706	0.711/0.334	0.638/0.498
Non-validated *r* ^2^/SEE	0.914/0.294	0.947/0.185	0.933/0.210
F	104.511	139.423	121.534
*r* ^2^_bootstrap_	0.916±0.023	0.965±0.010	0.928±0.015
S_bootstrap_	0.285±0.098	0.152±0.071	0.231±0.135
Optimal components	6	6	6
Field distribution%			
Steric	45.0	47.9	16.0
Electrostatic	55.0	52.1	25.2
Hydrophobic			18.9
H-bond Donor			10.1
H-bond Acceptor			29.8

**Table 2:** The actual pIC_50_, predicted pIC_50_ (Pred.) and their residuals (Res.) of the training and test set molecules.

Compd.	pIC_50_	CoMFA		CoMSIA	
No.	Actual	Pred.	Res.	Pred.	Res.
**1**	6.027	5.905	−0.122	6.329	0.302
**2**	6.108	6.099	−0.009	6.005	−0.103
**3**	6.721	6.801	0.080	6.579	−0.142
**4** *	6.770	6.568	−0.102	6.556	−0.114
**5**	6.638	6.715	0.077	6.734	0.096
**6**	5.921	5.893	−0.028	6.210	0.289
**7**	7.523	7.482	−0.041	7.601	0.078
**8**	8.000	7.907	−0.093	7.693	−0.307
**9**	7.509	7.605	0.096	7.309	−0.200
**10**	8.398	8.474	0.076	8.511	0.113
**11**	8.699	8.759	0.060	8.559	−0.140
**12**	8.699	8.736	0.037	8.368	−0.331
**13**	7.398	7.411	0.013	7.223	−0.175
**14**	6.456	6.806	0.350	6.931	0.475
**15** *	7.398	7.189	−0.209	7.098	−0.300
**16**	7.523	7.205	−0.318	7.167	−0.356
**17** *	7.097	7.089	−0.008	7.193	0.096
**18**	7.523	7.401	−0.122	7.347	−0.176
**19**	6.886	7.209	0.323	6.400	−0.486
**20**	7.000	6.890	−0.110	7.208	0.208
**21** *	7.222	6.800	−0.422	7.377	0.155
**22** *	8.000	7.823	−0.177	7.633	−0.367
**23**	8.097	7.907	−0.190	7.911	−0.186
**24** *	7.699	7.409	−0.260	7.205	−0.494
**25**	5.397	5.517	0.120	5.591	0.194
**26** *	5.000	5.299	0.299	5.394	0.394
**27**	5.482	5.478	−0.004	5.389	−0.093
**28**	5.854	5.726	−0.128	5.898	0.044
**29** *	6.319	6.820	0.501	6.907	0.588
**30**	6.854	6.703	−0.151	7.017	0.163
**31**	4.959	5.028	0.069	4.784	−0.175
**32**	5.791	6.134	0.343	6.132	0.341
**33**	6.409	6.632	0.223	6.703	0.294
**34**	6.482	6.539	0.057	6.192	−0.290
**35** *	6.658	6.605	−0.053	6.523	−0.135
**36**	7.000	6.898	−0.102	6.749	−0.251
**37**	6.886	6.769	−0.117	7.007	0.121
**38**	5.638	5.700	0.062	6.094	0.456
**39**	5.959	5.780	−0.179	6.043	0.084
**40** *	5.602	5.460	−0.142	5.950	0.348
**41** *	5.699	5.711	0.012	5.901	0.202
**42**	4.674	4.597	−0.077	4.406	−0.268
**43**	6.854	6.616	−0.238	7.010	0.156
**44**	5.456	5.502	0.046	5.512	0.056
**45**	6.000	6.003	0.003	6.237	0.237
**46**	6.036	6.104	0.068	5.989	−0.047
**47**	5.745	5.972	0.227	6.001	0.256
**48** *	6.495	6.502	0.007	6.618	0.123
**49**	7.432	7.207	−0.225	7.629	0.197
**50**	7.301	7.405	0.104	7.198	−0.103
**51**	6.886	6.789	−0.097	7.099	0.213
**52**	7.569	7.500	−0.069	7.613	0.044
**53**	7.387	7.237	−0.150	7.403	0.016
**54** *	6.886	7.066	0.180	7.100	0.214
**55**	4.801	4.834	0.033	4.748	−0.053
**56**	6.495	5.909	−0.586	6.001	−0.494
**57**	6.000	6.079	0.079	5.867	−0.133
**58**	5.553	5.604	0.051	5.713	0.160
**59** *	5.569	5.803	0.234	5.737	0.168
**60**	5.523	5.543	0.020	5.624	0.101
**61**	4.790	4.643	−0.147	4.996	0.206
**62**	6.432	6.507	0.075	6.670	0.238
**63**	6.357	6.503	0.146	6.609	0.252
**64**	7.699	7.499	−0.200	7.803	0.104
**65**	8.000	7.865	−0.135	7.787	−0.213
**66**	7.523	7.570	0.047	7.632	0.109
**67**	7.699	7.398	−0.301	8.004	0.305
**68**	7.523	7.723	0.200	7.422	−0.101
**69**	7.699	7.609	−0.090	7.865	0.166
**70**	5.886	6.003	0.117	5.599	−0.287
**71**	6.252	6.331	0.079	6.271	0.019
**72**	6.569	6.796	0.227	6.358	−0.211
**73**	7.222	7.301	0.079	6.933	−0.289
**74**	4.917	5.230	0.313	5.274	0.357
**75** *	6.886	6.558	−0.328	6.587	−0.299
**76**	6.959	6.960	0.001	6.789	−0.170
**77**	6.086	5.867	−0.219	6.372	0.286
**78**	7.097	7.109	0.012	6.903	−0.194
**79**	6.602	6.604	0.002	6.517	−0.085
**80**	5.903	5.913	0.010	6.043	0.140
**81**	5.108	5.140	0.032	5.006	−0.102

*Test set molecules.

The CoMSIA model comprising all five descriptors gave a *q^2^* value of 0.641 and *r* ^2^ value of 0.933 with an optimized component of 6, standard error estimate (SEE) of 0.210 and *F* value of 121.534. Contributions of steric, electrostatic, hydrophobic, hydrogen bond donor and acceptor fields were 0.160, 0.252, 0.189, 0.101 and 0.298, correspondingly.

### External Validation Results

The calculated results of the external validation were listed in Table **3**. For CoMFA and CoMSIA models, the calculated *r* ^2^_pred_ values were 0.875 and 0.769, with the slope (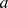) values of 1.021 and 1.201 (close to 1), intercept (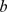) values of −0.025 and −0.039 (close to 0) and the correlation coefficient (R) values of 0.950 and 0.943 (close to 1), respectively. The valid *r* ^2^_m_ values of 0.669 and 0.631 (>0.5) as well as high slope of regression lines through the origin (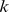) values of 0.986 and 0.991 (0.85≤k≤1.15) and the calculated [(*r^2^−r_0_^2^*)/*r^2^*] values of −0.079 and −0.100 (<0.1) were also obtained respectively. These external validation statistics revealed that both the CoMFA and CoMSIA models possessed high accommodating capacities and they would be reliable for predicting the pIC_50_ values of new derivatives.

**Table 3:** Results of the external validation for CoMFA and CoMSIA models.

Parameters	*r* ^2^_pred_	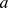 slope	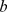 intercept	correlationcoefficient R	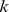 slope	*r* ^2^_m_	[(*r^2^−r_0_^2^*)/*r^2^*]
CoMFA	0.875	1.021	−0.025	0.950	0.986	0.669	−0.079
CoMSIA	0.769	1.201	−1.319	0.943	0.991	0.631	−0.100

### CoMFA Contour Maps

**Figure 5:**
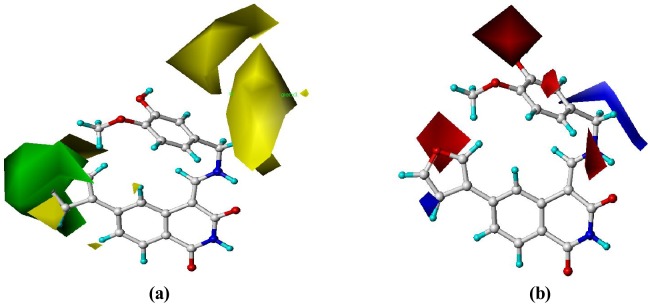
Std* coeff contour maps of CoMFA analysis in combination with compound 12. (**a**) Steric fields: green contours suggest regions where bulky groups increase activity, while yellow contours indicate regions where bulky groups decrease activity, and (**b**) Electrostatic fields: blue contours represent regions where electron-donating groups increase activity, while red contours highlight regions where electron-withdrawing groups increase activity.

Figure **5**. depicted the CoMFA steric and electrostatic contour plots for the most active compound **12**. For the steric field, the green contours represent regions of high steric tolerance (80% contribution) and the yellow contours (20% contribution) for unfavorable steric effect. The electrostatic field defined blue contours (80%) and red contours (20%) for electron-donating and -withdrawing substituents, respectively.

In Figure 5**a**, one huge green contour around the R_1_ position revealed that bulky substituents at this site would benefit the activity, and two huge yellow contours near the R_2_ and R_3_ positions suggested bulky groups at these sites unfavorable. This may explain the facts that derivatives **10–12** with relative bulkier groups (e.g. *N*-pyrrolyl, 3-furyl and 3-thienyl) at R_1_ displayed the strongest activity, while derivatives **25–28**, **31–32**, **38–42**, **44–45**, **47**, **55**, **57–60**, **74** and **80** bearing a relative bulkier substituents (e.g. 4-methylpiperazinyl, -CH_2_-(1-piperidinyl)) at R_3_ position showed a weak activity. Especially, derivatives **5**, **7** and **8** with the corresponding substituent of chloro, bromo and iodo showed their activities in the following order of **5**<**7**<**8**.

One red contour near the R_1_ and one red around the R_2_ and R_3_ in Figure 5**b** indicated an electron-withdrawing group favorable. The most potential derivatives **10–12** possessed corresponding electron-withdrawing aromatic groups (e.g. *N*-pyrrolyl, 3-furyl and 3-thienyl) at R_1_, while the activities of compounds **31–32**, **42** and **44–46** bearing electron-donating substituents (e.g. piperidinyl, -NHAc, morpholinyl, *N*,*N*-dimethylformamido-) decreased significantly. This also explained why compounds **1**, **3–5**, **7–16** and **19–24** with the corresponding methoxyl, hydroxyl, 2-furyl, 3-furyl, 3-pyridyl or 4-pyridyl group at R_3_ showed much more active than derivatives **25–60** and **74–75** with electron-donating substituent (1-piperidinylmethyl, substituted piperazinyl). A blue contour around the N-10 and C-11 positions emphasized the extreme importance of the electron-donating aminomethyl group.

### CoMSIA Contour Maps

**Figure 6:**
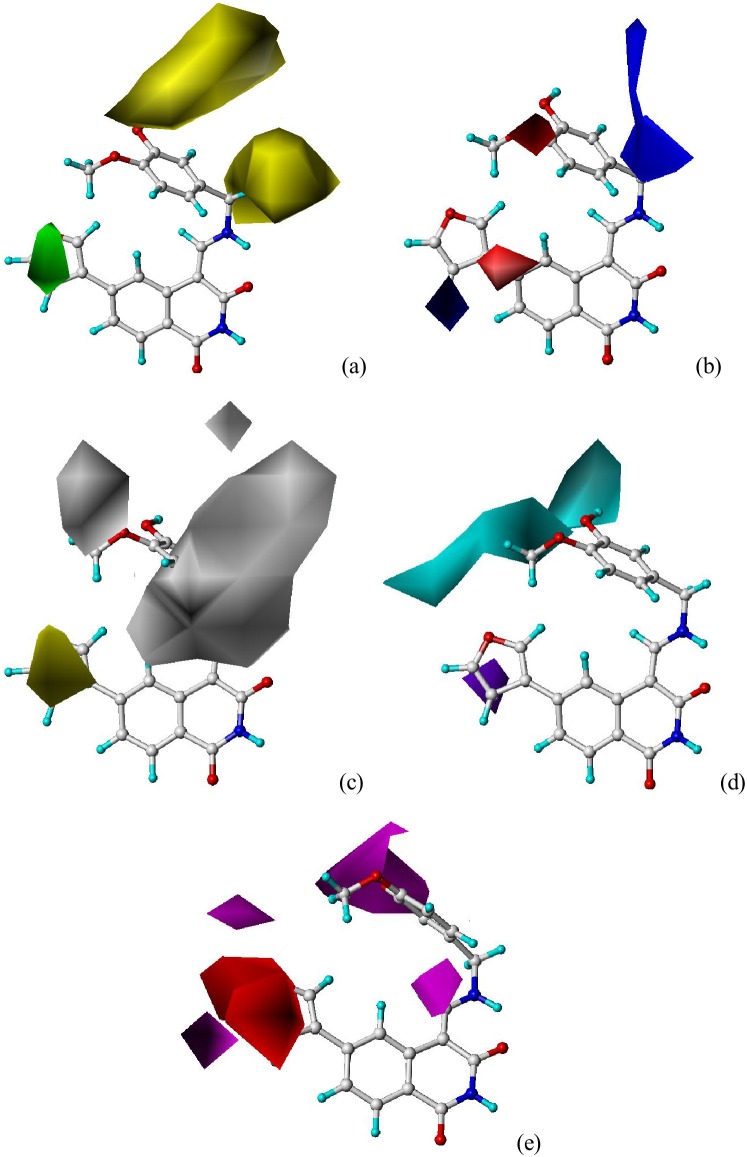
Std* coeff contour maps of CoMSIA in combination with compound 12. (**a**) Steric contour map. Green and yellow contours refer to sterically favored and unfavored regions. (**b**) Electrostatic contour map. Blue and red contours refer to regions where electron-donating and electron withdrawing groups are favored. (**c**) Hydrophobic contour map. White and yellow contours represent regions where hydrophilic and hydrophobic substituent are favored. (**d**) Hydrogen bond donor contour map. The cyan and purple contours indicate favorable and unfavorable hydrogen bond donor groups. (**e**) Hydrogen bond acceptor contour map. The magenta and red contours demonstrated favorable and unfavorable hydrogen bond acceptor groups.

The CoMSIA steric, electrostatic, hydrophobic, hydrogen bond donor and acceptor contours plots for the compound **12** were shown in Figure **6**. The CoMSIA steric and electrostatic contour maps (Figs. **6a and 6b**) were similar as the CoMFA steric and electrostatic contour maps (Figure **5**). For hydrophobic field, white (20% contribution) and yellow (80% contribution) contours highlighted hydrophilic and hydrophobic properties, respectively. Hydrogen bond donor and acceptor fields take the cyan (80%) and purple (20%) contours for hydrogen bond donor, and the magenta (80%) and red (20%) contours as favor and unfavor for hydrogen bond acceptor, correspondingly.

In Figure 6**c**, one yellow contour near the R_1_ region demonstrated that a hydrophobic substituent at this site would benefit the activity. Most of the active derivatives involved in present study possessed a hydrophobic group (e.g. 3-furyl, *N*-pyrrolyl, 3-thienyl, bromo-, chloro-, iodo-, phenyl) at R_1_, while those with only a hydrogen atom (e.g. **2**, **3**, **6** and **25–27**) exhibited significantly decreased potencies. Three pieces of white contour around the R_2_ position highlighted the hydrophilic properties of compounds **1–25** and **62–69**. One purple contour near the R_1_ position in Figure 6d suggested a hydrogen bond donor group unfavorable. Therefore, the compounds **10–12**, **49**, **50** and **52–53** with hydrogen bond acceptor oxygen or nitrogen atoms at R_1_ site exhibited better potencies. Both one red and two magenta contours located near the R_1_ position in Figure **6e** revealed that the hydrogen bond acceptor field was not very important for this site. The magenta contour near the R_2_ and R_3_ sites indicated hydrogen bond acceptor properties favorable. The hydroxyl groups at R_2_ could act as hydrogen bond acceptor at the same time. Therefore, the magenta contour confirmed the importance of the hydroxyl group at this region. Compounds **7–12** and **23** bearing a hydrogen bond acceptor substituent (methoxyl, 4-pyridyl) at R_3_ showed the most activities.

### Molecular Docking Analysis

**Figure 7:**
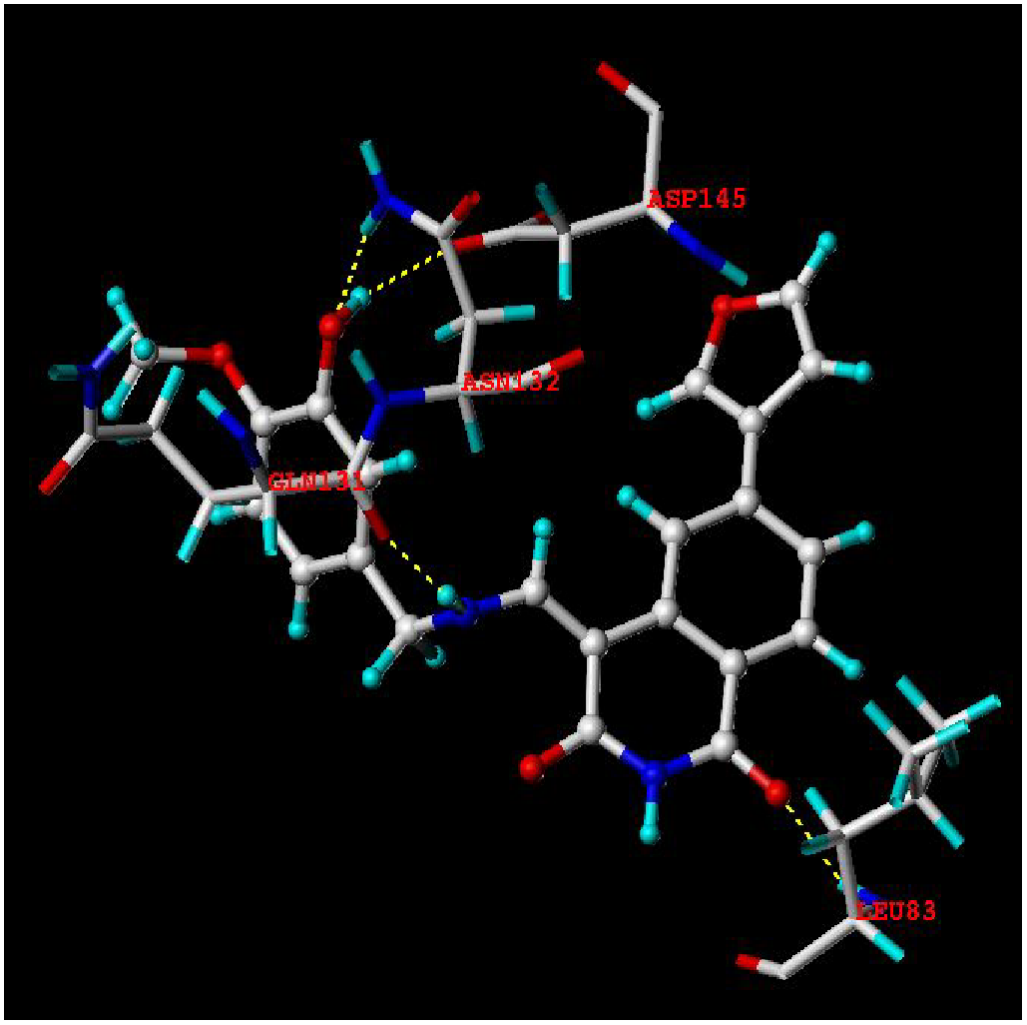
The binding mode between selected compound 12 and the ATP pocket of CDK4 (PDB code: 1GIH).

Figure **7**. illustrated the binding modes between compound **12** and the ATP pocket. The carbonyl group at C-3 position acted as a hydrogen bond acceptor by forming a H-bond with the -NH group of Leu83 residue; the imino group at N-10 position served as a hydrogen bond donor and formed H-bond with the carbonyl group of Gln131 residue; the hydroxyl group at R_2_ site acted as both hydrogen bond donor and acceptor and formed two H-bonds with the carbonyl group of Asp145 and the -NH group of Asn132 residues, respectively. The observations taken from Figure **7** satisfactorily matched the corresponding CoMSIA hydrogen bond donor and acceptor contour maps.

### Summary of Structure-activity Relationship

**Figure 8:**
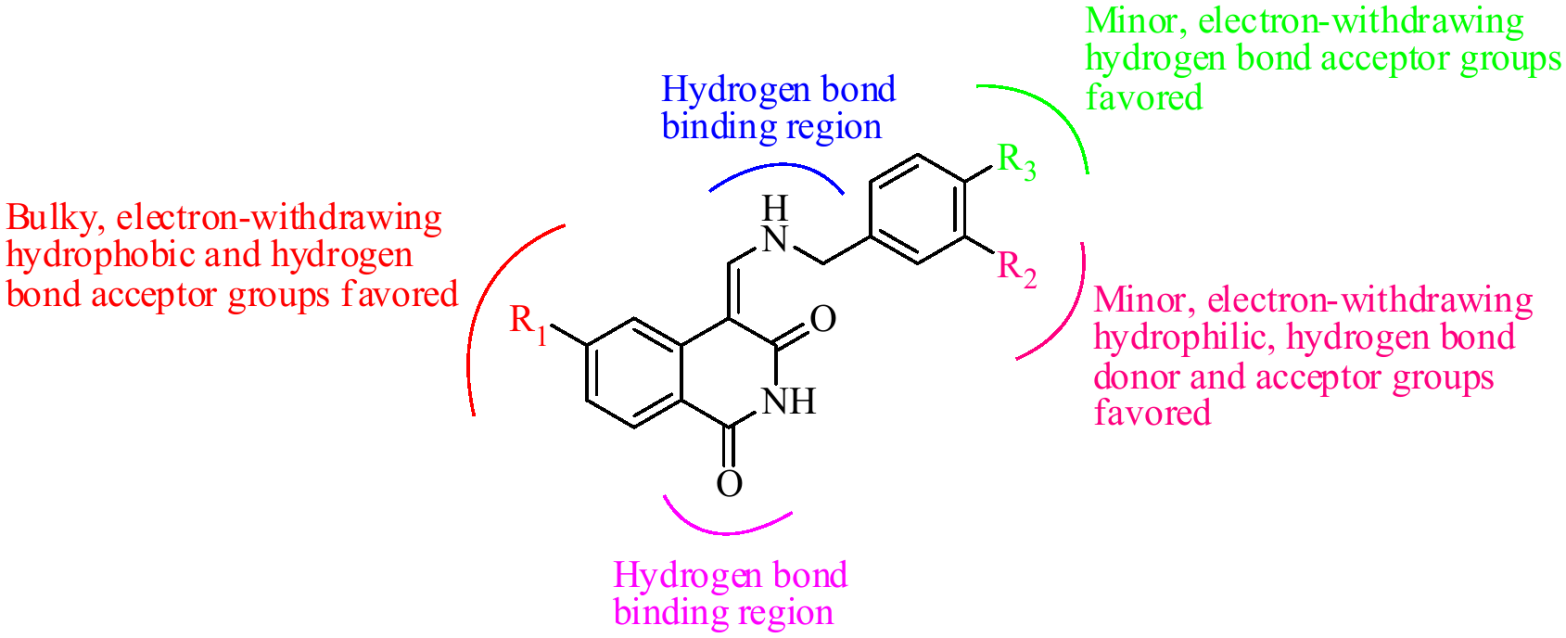
Structure-activity relationship revealed by 3D-QSAR and docking studies.

The structure-activity relationship revealed by 3D-QSAR and docking studies was illustrated in Figure **8**. In short, the bulky, electron-withdrawing, hydrophobic and hydrogen bond acceptor groups at R_1_ position are favorable; the minor, electron-withdrawing, hydrophilic, hydrogen bond donor and acceptor groups at R_2_ position may benefit the potency; the minor, electron-withdrawing and hydrogen bond acceptor substituent at R_3_ position would increase the activity. The carbonyl group at C-3 and the imino group at N-10 site were essential for binding to the ATP pocket of CDK4.

### Designing of Potent Derivatives

**Figure 9:**
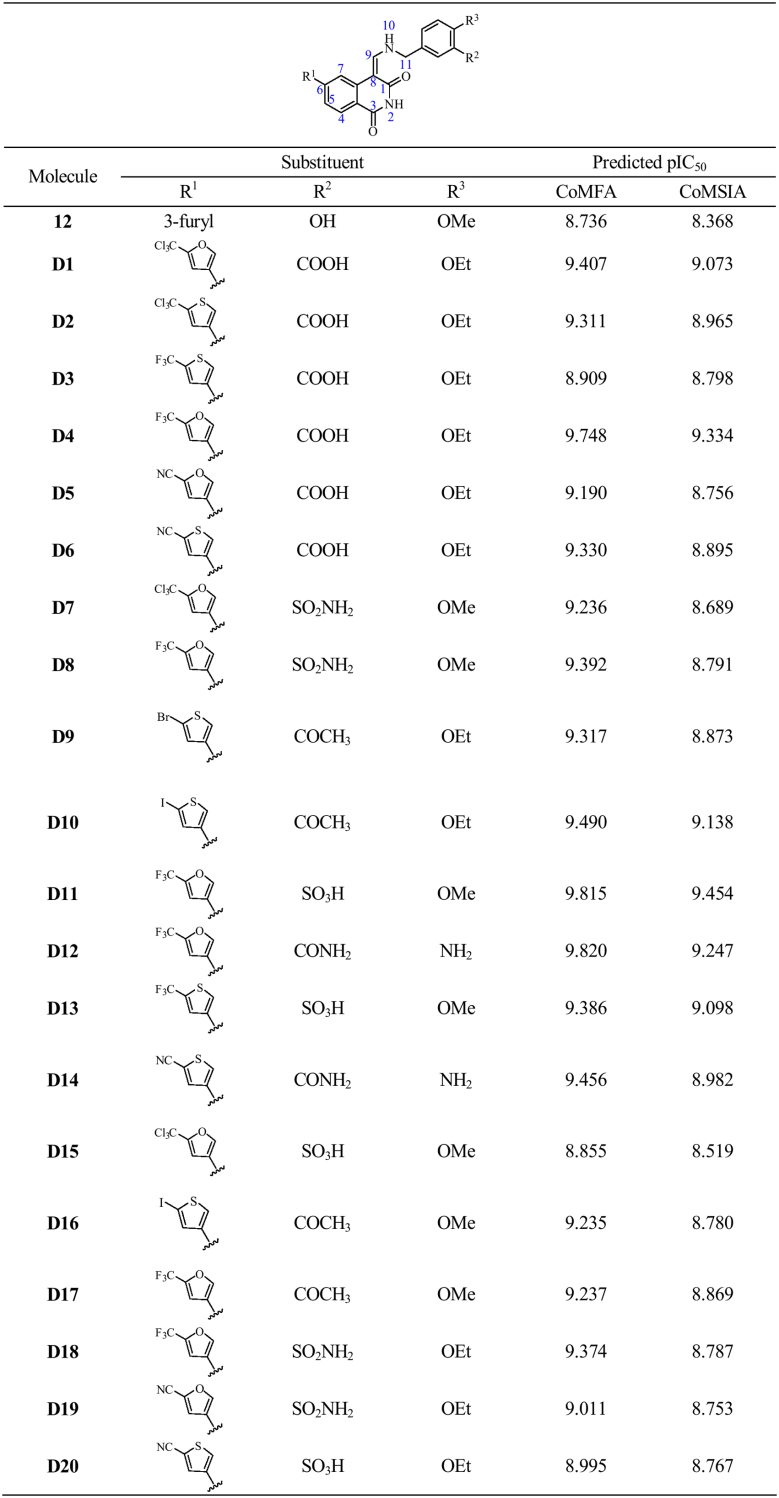
Structures and predicted pIC50 values of newly designed molecules.

Based on the structure-activity relationship revealed by the present study, twenty novel isoquinoline- 1, 3-(2*H*,4*H*)-dione derivatives were designed. These molecules were aligned to the database and their activities were predicted better than compound **12** by the best CoMFA and CoMSIA models established previously, especially **D4**, **D11** and **D12** showed 10 folds more active than compound **12**. The chemical structures and predicted pIC_50_ values of these compounds were shown in Figure **9**. The results validated the structure-activity relationship in this present work.

## Conclusions

In this frame-work, a combined docking and 3D-QSAR analysis was performed to explore the interactions between isoquinoline-1,3-diones and CDK4 protein. The satisfactory CoMFA model (*q* ^2^ = 0.695, *r* ^2^ = 0.947) and CoMSIA model (*q* ^2^ = 0.641, *r* ^2^ = 0.933) showing good correlative and predictive abilities were obtained via internal and external cross-validation techniques, region focusing, bootstraping and leave-group-out. Our analyses found that all five parameters (steric, electrostatic, hydrophobic, hydrogen bond donor and acceptor properties) are highly desirable for potent inhibitory activity. The contour maps and the docking binding structures showed that the carbonyl group at C-3 and the imino group at N-10 site were necessary for binding to the ATP pocket of CDK4. Based on the interactions, twenty new designed molecules predicted higher activities than compound **12**, confirming that the models could provide a valuable clue for the development of more active CDK4 subtype-selective inhibitors.
